# Aseptic Meningitis as First Presentation of Acute Myeloid Leukemia With Normal Cell Blood Count

**DOI:** 10.1002/ccr3.71132

**Published:** 2025-10-12

**Authors:** Mahnaz Arian, Abolghasem Allahyari, Marzieh Kazerani, Ahmadreza Zarifian, Kiana Ketabi, Hossein Alavi, Melika Farshidianfar

**Affiliations:** ^1^ Department of Infectious Diseases, Faculty of Medicine Mashhad University of Medical Sciences Mashhad Iran; ^2^ Hematology–Oncology Department, Faculty of Medicine Mashhad University of Medical Sciences Mashhad Iran; ^3^ Department of Infectious Diseases MMS.C., Islamic Azad University Mashhad Iran; ^4^ Cellular Pathology Department St. Georges University Hospital London UK; ^5^ Faculty of Medicine Mashhad University of Medical Sciences Mashhad Iran; ^6^ Family Medicine Department, Schulich School of Medicine and Dentistry Western University London Ontario Canada

**Keywords:** acute, aseptic, blood cell count, leukemia, meningitis, myeloid

## Abstract

Aseptic meningitis can be the initial manifestation of acute myeloid leukemia (AML), even when the complete blood count (CBC) is normal. A single cerebrospinal fluid (CSF) analysis for malignant cells may be insufficient and could delay diagnosis. Therefore, infections, collagen vascular diseases, and malignancies—particularly leukemias—should be carefully considered.

## 1 Introduction

AML is the most common type of leukemia in adults. It can involve both the bone marrow and extramedullary sites. CNS involvement may be asymptomatic or present with symptoms such as headaches, lethargy, seizures, cranial nerve palsies, or hemorrhage, and is associated with a poor prognosis [1]. Aseptic meningitis has multifactorial etiologies, including both infectious and noninfectious causes. Increasing awareness of these causes is crucial for physicians [2]. We report a 22‐year‐old patient in whom aseptic meningitis was the initial manifestation of AML with a normal CBC and differential count.

## 2 Case History/Examination

A 22‐year‐old male farmer was admitted with severe headaches, seizures, fever, chills, nausea, and vomiting. He presented with a 2‐month history of persistent headaches radiating to the orbit, along with recurrent seizures. He received empirical treatment with doxycycline and streptomycin despite negative laboratory tests for brucellosis. He came to the emergency department with a high fever of 39.3°C, a blood pressure of 130/80 mmHg, a heart rate of 100 beats per minute, and symptoms of meningitis, including neck stiffness and positive Brudzinski's and Kernig's signs. White blood cell (WBC) count was 6900/μL, with 83.3% polymorphonuclears (PMNs). The red blood cell (RBC) count was 4.3 million/μL, the hemoglobin level was 12.8 g/dL, and the mean corpuscular volume (MCV) was 79.9 fl. The C‐reactive protein (CRP) level was 121 mg/L. Wright and 2‐mercaptoethanol (2‐ME) brucella agglutination tests were negative. He had hypoalbuminemia (2.7 g/dL). The computerized tomography (CT) scan of the brain was normal. The cerebrospinal fluid (CSF) glucose level of 48 mg/dL, protein level of 262 mg/dL, and WBC count of 820/μL (80% PMNs), with no RBCs detected. The CSF polymerase chain reaction (PCR) for herpes simplex virus was negative. He was treated empirically for bacterial meningitis with vancomycin (1 g every 12 h) and ceftriaxone (2 g every 12 h) based on his CSF findings, but this treatment did not lead to improvement. The analysis of the second lumbar puncture (LP) showed a glucose level of 42 mg/dL, a protein level of 152 mg/dL, and a WBC count of 6900/μL with 62% lymphocytes and 38% PMNs. Based on these findings, empirical treatment for tuberculosis (TB) was started with isoniazid, rifampin, pyrazinamide, and ethambutol, along with adjunctive corticosteroids to reduce inflammation. The lack of improvement with the anti‐TB regimen prompted consideration of fungal or autoimmune conditions. A third LP at the end of treatment revealed a WBC of 110/μL (90% lymphocytes and 10% neutrophils), a protein level of 10 mg/dL, and a glucose level of 40 mg/dL. CSF and blood cultures remained negative throughout admission. As the patient's headaches persisted and he reported a history of recurrent oral aphthous ulcers, a rheumatology consultation was performed. Following a positive Pathergy test, the patient was diagnosed with Behçet's syndrome and started on treatment with prednisolone (5 mg three times daily) and cyclosporine before being discharged.

**FIGURE 1:**
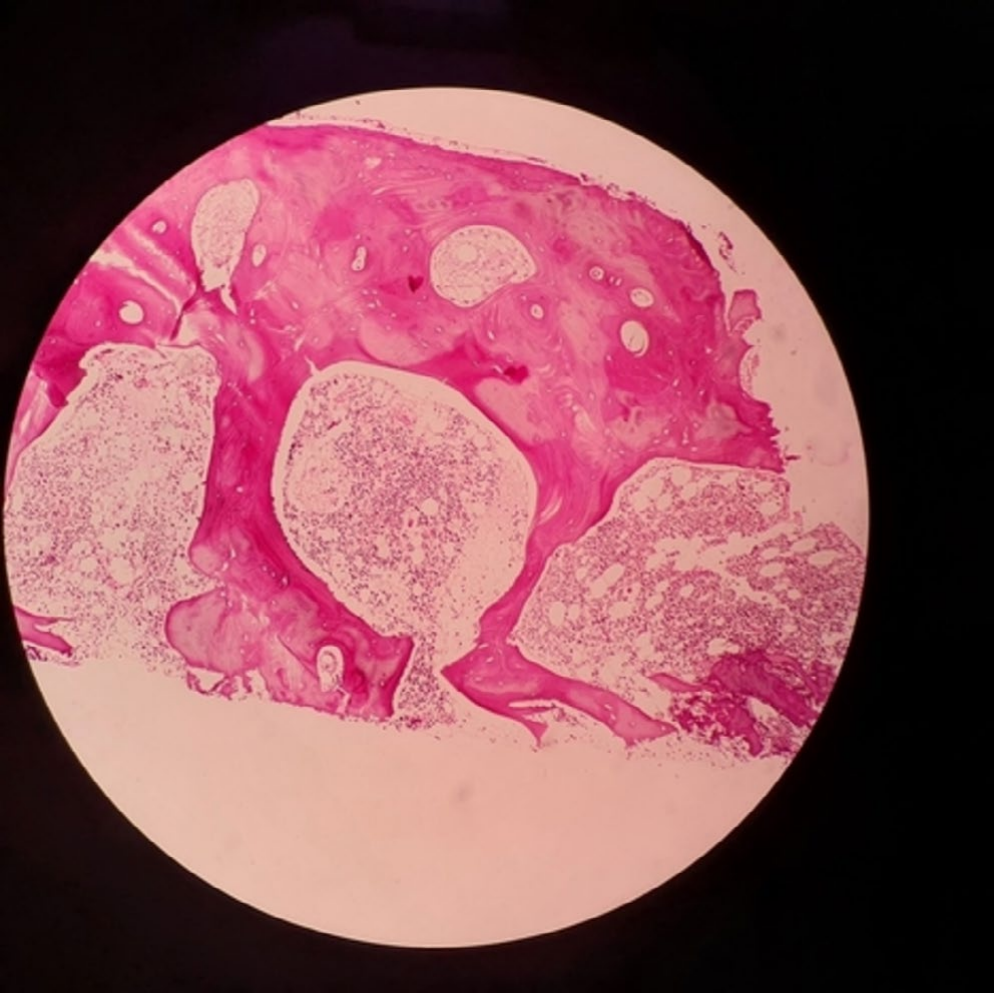
Bone marrow stained with hematoxylin and eosin.

**FIGURE 2:**
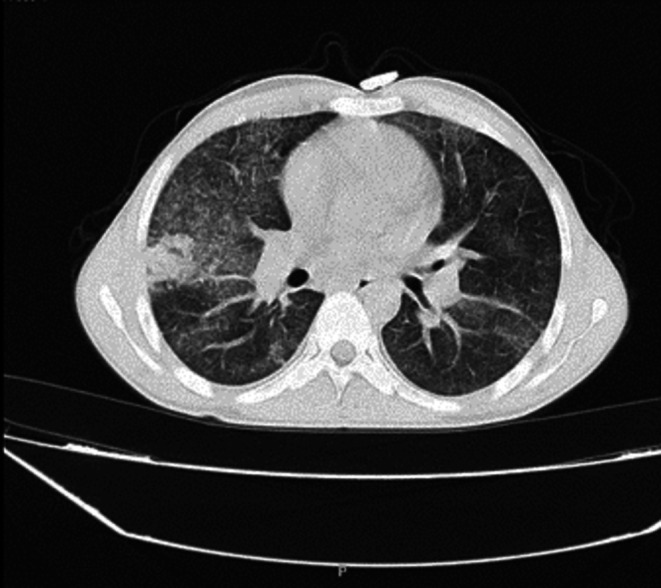
HRCT showed pulmonary infection.

**FIGURE 3:**
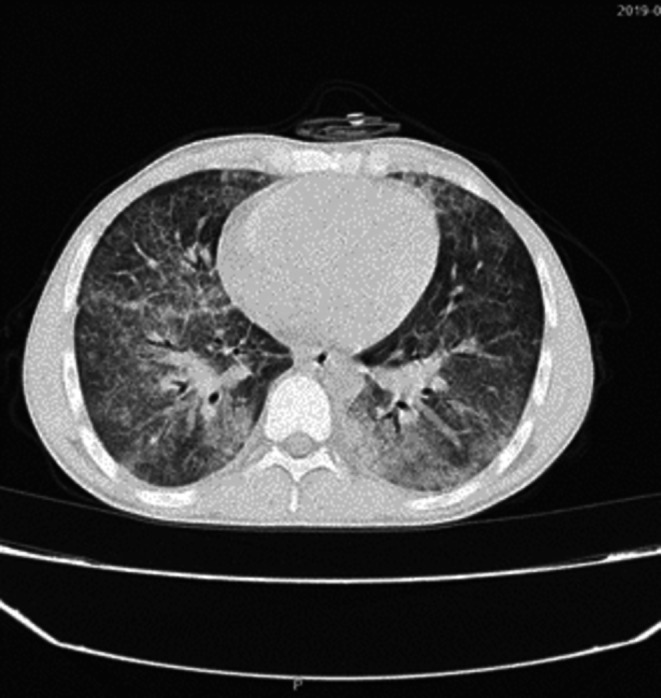
Axial chest CT scan showing bilateral ground‐glass opacities and interstitial thickening.

He returned 1 month later with a headache, generalized seizures, and signs of meningism, including positive Brudzinski's and Kernig's signs. In the fourth LP, he had 2200 WBC/μL (47% PMN, 53% lymphocyte), with a protein level of 382 mg/dL and a glucose level of 48 mg/dL. Empirical treatment with ceftriaxone, vancomycin, and mannitol was initiated. CSF PCR tests for TB and cryptococcus were negative. Blood cultures (two sets), CSF culture, human immunodeficiency virus (HIV) tests, complement levels (CH50, C3, and C4), and Borrelia tests were also negative. The abdominal and pelvic ultrasound, CT scan of the paranasal sinuses, and chest X‐ray were normal. Although vertigo and imbalance were observed, all vestibulocochlear examinations were normal. Magnetic resonance venography (MRV) and contrast‐enhanced brain and spine magnetic resonance imaging (MRI) also showed normal results. Eventually, the fourth CSF analysis (Table 1) revealed atypical lymphocytes, and flow cytometry of CSF and bone marrow aspiration, the patient was diagnosed with AML (Figure 1). He was subsequently transferred to the hematology department, where he received systemic and intrathecal chemotherapy. While awaiting bone marrow transplantation, he developed a severe pulmonary infection (Figures 2 and 3), which progressed despite intensive treatment, ultimately resulting in septic shock and death.

**TABLE 1:** Changes in CSF.

WBC	PMN	Lymph	Glucose	Protein
820/μL	80%	20%	48/mg/dL	262/mg/dL
6900/μL	38%	62%	42/mg/dL	152/mg/dL
110/μL	10%	90%	40/mg/dL	10/mg/dL
2200/μL	47%	53%	48/mg/dL	382/mg/dL

## 3 Differential Diagnosis, Investigations, and Treatment

Differential diagnoses include acute bacterial meningitis, brucellosis, TB meningitis, Behçet's syndrome, cryptococcal meningitis, HIV, and malignancy.

## 4 Discussion

AML can present with a wide range of clinical symptoms including cytopenia, leukocytosis, infections, bleeding, and disseminated intravascular coagulation (DIC), which are influenced by genetic and epigenetic variations [3]. The diagnosis is usually confirmed when 20% or more blasts are present in the bone marrow or peripheral blood [4]. AML may also involve extramedullary sites outside the bone marrow and blood, which is often linked to a poorer prognosis [1]. CNS involvement in AML can occur either directly through neoplastic infiltration of leukemic cells or indirectly due to thrombotic or hemorrhagic complications caused by thrombocytopenia, hypercoagulability, and hyperleukocytosis [5]. The incidence of CNS involvement in AML is from 0.6% to 3% with a recent study reporting that only 0.6% of patients experienced symptoms at diagnosis [6]. CNS manifestations of AML include headache, imbalance, fainting, mood changes, nausea, vomiting, seizures, cranial nerve palsies (especially the 3rd, 5th, 6th, and 7th cranial nerves), and papilledema. However, most patients remain asymptomatic, often diagnosed only through paraclinical tests or postmortem examinations. Due to its atypical presentation and urgent nature, diagnosing CNS AML poses significant challenges to clinicians. Diagnosis of CNS AML is based on clinical signs, cytological analysis, flow cytometry of cerebrospinal fluid, and neuroimaging [7]. Although detecting blast cells in the CSF can aid in diagnosis, caution is necessary as there might be false‐positive results. Interestingly, a study by Siegal et al. stated that CNS AML may be induced iatrogenic due to procedures like LP, chemotherapy, and stem cell transplantation [5].

Men with AML are three times more likely than women to have CNS involvement, and this complication is typically seen in younger patients, which aligns with the case we presented. Chromosomal abnormalities such as trisomy 8 and chromosome 16 inversion, as well as elevated lactate dehydrogenase (LDH) levels and low serum lysozyme levels have been associated with the risk of CNS involvement [8]. However, the patient exhibited low LDH levels in our study, and we did not perform chromosomal karyotyping. The presence of hyperleukocytosis (100,000 leukocytes per microliter) and the expression of the Ceramide‐Dodecasaccharide (CD)‐56 adhesion molecule are also associated with CNS involvement in AML [6]. While fewer than 5% of AML patients have a normal blood count, our patient showed a normal CBC, which caused challenges and delays in diagnosis [4].

CT and MRI are valuable diagnostic tools, and contrast‐enhanced MRI is generally the preferred modality [6, 8]. Nevertheless, half of cases remain undiagnosed, even with detailed MRI assessments, which makes the diagnosis more challenging [6, 8]; similarly, in the present study, MRI and MRV showed no abnormalities. Our patient had normal WBC, LDH, along with one negative CSF cytology test, which led to a delay in diagnosis. We must notice that low sensitivity CSF cytology [9] and malignant cells do not have a homogeneous distribution in cerebrospinal fluid. We should explore more accurate techniques for the timely diagnosis of CNS AML. Detection of blast cells in CSF is often difficult, and ancillary methods such as CSF levels of sL‐selectin or interleukin‐6 are helpful in the diagnosis of CNS involvement in AML [10].

In conclusion, CNS AML is an uncommon complication with a poor prognosis. Although CSF cytology and flow cytometry may be important in diagnosing associated meningitis, more sensitive and specific tests for CSF analysis are needed, as early diagnosis and treatment are essential for improving outcomes.

## Author Contributions

**Mahnaz Arian:** conceptualization, supervision. **Abolghasem Allahyari:** investigation, validation. **Ahmadreza Zarifian:** data curation, visualization. **Kiana Ketabi:** software, visualization. **Hossein Alavi:** project administration. **Melika Farshidianfar:** investigation, writing – original draft. **Marzieh Kazerani:** writing – original draft, writing – review and editing.

## Ethics Statement

Patient anonymity has been preserved, and consent for publication has been obtained from the patient.

## Consent

A written informed consent was obtained from the patient for the publication of this clinical case report. The patient's consent was given in accordance with the journal's patient consent policy, ensuring that all identifying information has been anonymized to maintain confidentiality.

## Conflicts of Interest

The authors declare no conflicts of interest.

## Data Availability

The data that support the findings of this study are available from the corresponding author upon reasonable request.
